# Potential Relation between Plasma BDNF Levels and Human Coronary Plaque Morphology

**DOI:** 10.3390/diagnostics11061010

**Published:** 2021-06-01

**Authors:** Patrizia Amadio, Nicola Cosentino, Sonia Eligini, Simone Barbieri, Calogero Claudio Tedesco, Leonardo Sandrini, Marta Zarà, Franco Fabiocchi, Giampaolo Niccoli, Giulia Magnani, Francesco Fracassi, Filippo Crea, Fabrizio Veglia, Giancarlo Marenzi, Silvia Stella Barbieri

**Affiliations:** 1 Unit of Brain-Heart Axis: Cellular and Molecular Mechanisms, Centro Cardiologico Monzino, IRCCS, 20138 Milan, Italy; patrizia.amadio@ccfm.it (P.A.); leonardo.sandrini@ccfm.it (L.S.); marta.zara@ccfm.it (M.Z.); 2 Intensive Cardiac Care Unit, Centro Cardiologico Monzino, IRCCS, 20138 Milan, Italy; nicola.cosentino@ccfm.it (N.C.); giancarlo.marenzi@ccfm.it (G.M.); 3 Unit of Metabolomics and Cellular Biochemistry of Atherothrombosis, Centro Cardiologico Monzino, IRCCS, 20138 Milan, Italy; sonia.eligini@ccfm.it; 4 Unit of Biostatistics, Centro Cardiologico Monzino, IRCCS, 20138 Milan, Italy; barbieri.simo@gmail.com (S.B.); calogero.tedesco@ccfm.it (C.C.T.); fabrizio.veglia@ccfm.it (F.V.); 5 Interventional Cardiology Unit, Centro Cardiologico Monzino, IRCCS, 20138 Milan, Italy; franco.fabbiocchi@ccfm.it; 6 Cardiology Unit, Department of Medicine and Surgery, University of Parma, 43121 Parma, Italy; giampaolo.niccoli@unipr.it (G.N.); giulia.magnani@unipr.it (G.M.); 7 Department of Cardiovascular and Thoracic Sciences, Catholic University of the Sacred Heart, Fondazione Policlinico Universitario Agostino Gemelli-IRCCS, 00168 Rome, Italy; francesco.fracassi@yahoo.it (F.F.); Filippo.Crea@unicatt.it (F.C.)

**Keywords:** BDNF, plaque morphology, plaque vulnerability, OCT, CAD, stable angina, acute myocardial infarction

## Abstract

Coronary artery disease (CAD) patients are at high ischemic risk, and new biomarkers reflecting atherosclerotic disease severity and coronary plaque vulnerability are required. The Brain-Derived Neurotrophic Factor (BDNF) affects endothelial and macrophage activation suggesting its involvement in atherosclerotic plaque behavior. To investigate whether plasma BDNF is associated with in vivo coronary plaque features, assessed by optical coherence tomography (OCT), in both acute myocardial infarction (AMI) and stable angina (SA) patients, we enrolled 55 CAD patients (31 SA and 24 AMI), and 21 healthy subjects (HS). BDNF was lower in CAD patients than in HS (*p* < 0.0001), and it decreased with the presence, clinical acuity and severity of CAD. The greater BDNF levels were associated with OCT features of plaque vulnerability in overall CAD as well as in SA and AMI patients (*p* < 0.03). Specifically, in SA patients, BDNF correlated positively with macrophages’ infiltration within atherosclerotic plaque (*p* = 0.01) and inversely with minimal lumen area (*p* = 0.02). In AMI patients a negative correlation between BDNF and cap thickness was found (*p* = 0.02). Despite a small study population, our data suggest a relationship between BDNF and coronary plaque vulnerability, showing that vulnerable plaque is positively associated with plasma BDNF levels, regardless of the clinical CAD manifestation.

## 1. Introduction

Coronary artery disease (CAD) is the main cause of death in both developed and developing countries [1]. Patients with CAD, and in particular those with acute myocardial infarction (AMI), are at high risk of major adverse cardiovascular events, despite the adherence to current guideline-recommended secondary prevention therapies [2,3,4,5,6]. Their persistent high ischemic risk highlights the need to have new biomarkers reflecting atherosclerotic disease severity and coronary plaque activity and vulnerability.

Brain-derived neurotrophic factor (BDNF) belongs to the family of neurotrophins that are well characterized as trophic factors for neurons. Several studies have also demonstrated their key role in non-neuronal cells, such as endothelial cells, smooth muscle cells, immune cells and epithelial cells in different organs [7,8]. The pleiotropic effects of this neurotrophin, ranging from neuroplasticity to angiogenesis, also include vascular integrity [9] and fibrin clot stability [10].

Circulating BDNF levels have been previously related to cardiovascular disease, and several studies have recently suggested its prognostic relevance in patients with hypertension [11], diabetes mellitus [12], Chagas’ cardiomyopathy [13], heart failure [14], and CAD [15]. In particular, elevated BDNF levels have been associated with decreased risk of coronary events and mortality [15], while, reduced BDNF levels have been detected in CAD and in heart failure patients [16,17], and related to endothelial function [18].

However, its relationship with atherosclerosis is still controversial. Indeed, high BDNF expression has been detected in the atheromatous intima and adventitia of atherosclerotic arteries, as well as in smooth muscle cells, macrophages, and fibroblasts of human and mouse atherosclerotic plaques [19], suggesting a potential role of BDNF in coronary atherosclerosis progression and plaque activation and instability. In vitro studies have provided evidence that BDNF enhances NAD(*p*)H oxidase activity with the generation of reactive oxygen species and promotes Tissue Factor activity in human coronary artery smooth muscle cells and human monocytes [19,20], indicating a possible relationship among these type of cells, BDNF and atherosclerotic plaque activity. In addition, circulating levels of BDNF reflect the platelet activity and the inflammatory status [21,22,23]. Despite all these conflicting results, there are no studies investigating the relationship between circulating BDNF and in vivo morphological characteristics of coronary plaques.

Optical coherence tomography (OCT) analysis is an in vivo intravascular imaging modality that provides high-resolution (10 µm) images of the atherosclerotic plaque, providing detailed information about its morphology and composition [24]. Taking advantage of the OCT images of the atherosclerotic plaque, it has been recently shown that serum BDNF levels are independently associated with the presence of intraplaque macrophages in patients with AMI [25].

Here, we aimed at assessing whether plasma BDNF levels are associated with coronary plaque features in AMI patients and in those with stable angina (SA), with the goal of pinpointing high-risk patients and vulnerable rupture-prone coronary plaques.

## 2. Materials and Methods

### 2.1. Study Design

The study was carried out at Centro Cardiologico Monzino, Milan, Italy. Fifty-five consecutive CAD patients undergoing coronary angiography due to SA (*n* = 31) or AMI (*n* = 24), as their first ischemic clinical presentation, and showing obstructive atherosclerosis (>50% diameter stenosis by visual estimate) at coronary angiography, were enrolled. In particular, patients with SA underwent coronary angiography due to a clinical history of typical angina during the exercise and evidence of at least moderate ischemia by stress test modality.

Twenty-one HS, with neither a history of CAD, clinical symptoms of myocardial ischemia, nor evidence of myocardial ischemia at stress test, and specifically not taking any cardiovascular therapy, were recruited as control group.

All CAD patients underwent coronary plaque composition analysis by OCT and measurement of plasma BDNF levels, while HS underwent BDNF evaluation only. The study was approved by the institutional Ethic Committee (Comitato Etico IRCCS-Istituto Europeo di Oncologia e Centro Cardiologico Monzino) (R21-CCM25), and was performed according to the Declaration of Helsinki. All the study subjects provided written informed consent at the time of enrollment.

### 2.2. Blood Collection, Plasma Preparation and BDNF Analyses

Blood was collected in HS at a scheduled visit, while in CAD patients it was collected within 18–24 h after hospital admission. Peripheral blood sample was collected into vacutainer tubes containing EDTA (ethylenediaminetetraacetic acid) disodium salt (9.3 mM; Vacutainer System, Becton Dickinson, Franklin Lakes, NJ, USA) and centrifuged within 30 min at 3000× *g* for 10 min at 4 °C. Plasma thus obtained was collected, aliquoted and immediately stored at −80 °C until analysis. BDNF was measured in plasma by the ELISA kit commercially available BDNF Emax Immunoassay system (Promega, Madison, WI, USA).

### 2.3. OCT Image Analysis

All the OCT measurements were performed as previously described [26]. Briefly, OCT analysis was performed at the MLA site of SA patients, while in AMI patients, culprit lesion was identified by angiography, electrocardiographic T-wave or ST-segment modifications, and/or regional wall motion abnormalities at echocardiogram. Frequency domain OCT (FD-OCT) images were obtained by a commercially available system (C7 System, LightLab Imaging Inc/St Jude Medical, Westford, MA, USA) connected to an OCT catheter (C7 Dragonfly; LightLab Imaging Inc/St Jude Medical). All images were digitally registered and saved, and two independent investigators, blinded to clinical and laboratory values, analyzed every single frame (0.2 mm) (Institute of Cardiology, Catholic University of the Sacred Heart, Policlinico Gemelli, Rome, Italy) [27]. As previously described [28,29,30], the analysis was focused on plaque characterization (calcified, fibrous, or lipid plaques), presence of plaque rupture, assessment of fibrous cap thickness, and presence of intracoronary thrombi and intra-plaque microchannels, all at the MLA site or at culprit lesion, in SA and AMI patients, respectively. If a plaque displayed two or more lipid-containing quadrants, it was considered a lipid-rich plaque, and the lipid arc and the cap thickness were measured, with TCFA defined as a lipid-rich plaque with a fibrous cap thickness of ≤65 μm [31].

### 2.4. OCT Macrophage Analysis

As previously reported [27], the presence of macrophage infiltration (MØI) was determined in the lesions, analyzed by OCT. Specifically, in accordance with the International Working Group for Intravascular Optical Coherence Tomography (IWG-IVOCT) Consensus standards [29], macrophages were qualitatively selected on raw OCT data within a 300 × 125 μm^2^ (lateral × axial) region of interest (ROI). Macrophages were visualized as signal-rich, distinct, or confluent punctate regions exceeding the intensity of background speckle noise and generating a backward shadowing. The depth of the ROI was matched to the cap thickness, for caps having a thickness <125 μm^2^. Median filtering was carried out with a 3 × 3 square kernel to remove speckle noise. NSD, known to have a high degree of positive correlation with histological measurements of macrophage infiltration, was employed for quantitative interpretation of macrophage content in plaques with MØI, through dedicated software provided by S. Jude Medical [30,31]. NSD was measured for each pixel within each cap using a 125 μm^2^ window centered at the pixel location: NSD (x, y) = [σ (x, y) 125 μm^2^/(Smax − Smin)] × 100, where NSD (x, y) is the normalized standard deviation of the OCT signal at pixel location (x, y), Smax is the maximum OCT image value, and Smin is the minimum OCT image value. Pixels within the (125 × 125) μm^2^ window that did not overlap with the segmented cap were excluded [30].

### 2.5. Plaque Vulnerability Assessment

In CAD patients plaque vulnerability index score was considered. In particular, TCFA, lipid plaque and MØI were the categorical parameters taken into consideration.

Patients were considered at low plaque vulnerability if they had none or only one of these parameters (group 0–1), and at high plaque vulnerability if they had two or all of them (group 2–3).

### 2.6. Statistical Analysis

Continuous variables were expressed as mean ± standard deviation (SD) or median with interquartile range, if they followed a normal or non-normal distribution, respectively. Continuous variables were compared with unpaired t-test or Mann-Whitney U-test, whereas categorical variables were compared using the Chi-square test or Fisher’s exact test, as appropriate. All analyses were also adjusted for age and sex, and for prior treatment (Statin, Aspirin, β-blockers and ACE-inhibitors) or for cardiovascular risk factors (smoke, diabetes, hypertension, dyslipidemia and familiarity), where indicated. Correlations between variables were performed using the Spearman’s rank coefficient to account for possible non-linear associations. Partial correlations adjusted for age sex and AMI presentation were also computed. Intra-observer and inter-observer variability in the analysis of MØI in the fibrous cap were assessed by Kappa measure of agreement. Regarding the evaluation of MØI, Kappa measures of agreement for intra-observer and inter-observer variability were 0.91 (*p* < 0.0001) and 0.95 (*p* < 0.001), respectively. As BDNF measurements were not normally distributed the values were log-transformed before comparison among groups. All tests were two-sided. A *p* value < 0.05 was considered to indicate statistical significance. All calculations were computed by SAS software package (Version 9.2; SAS Institute Inc., Cary, NC, USA).

## 3. Results

### 3.1. Clinical Characteristics and Plasma BDNF Levels in the Study Population

In the present study, we enrolled 55 CAD and 21 healthy subjects (HS). The demographic, clinical, and laboratory features of the study population, as well as prior pharmacological treatment, are listed in Table 1.

Patients with CAD had lower total and low-density lipoprotein cholesterol levels (LDL) (*p* < 0.01, and *p* < 0.001, respectively) than HS, as 34.5% of CAD patients were on chronic statin (*p* = < 0.0001). Moreover, they had higher white blood cells count (*p* < 0.0001), glycaemia (*p* = 0.0003), and hemoglobin (*p* < 0.0001).

**Figure 1:**
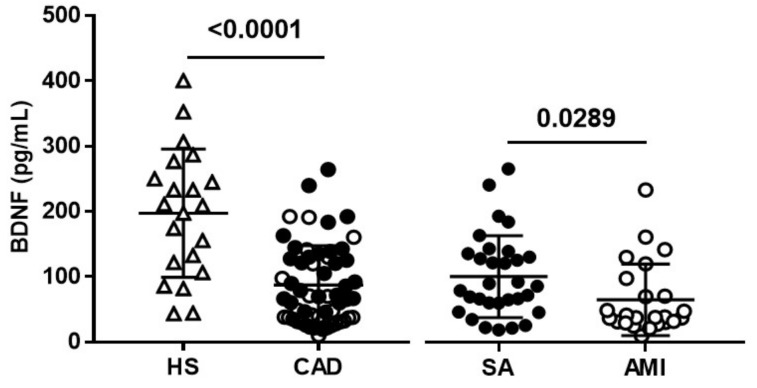
Plasma BDNF levels. BDNF was measured in healthy subjects (HS) and coronary artery disease (CAD) patients with diagnosis of stable angina (SA) and acute myocardial infarction (AMI). Data are expressed as mean  ±  SEM.

As previously shown [32], patients with CAD had significant lower plasma BDNF levels compared to HS (69.66 (10.0–264.4) pg/mL versus 208.9 (43.5–400.5) pg/mL, *p* < 0.0001) (Figure 1), and this difference was also maintained after adjustment for prior treatment and cardiovascular risk factors (*p* < 0.0005).

Of the 55 CAD patients, 31 (56%) had a diagnosis of SA and 24 (44%) of AMI. Table S1 summarizes major clinical and laboratories characteristics of SA and AMI patients.

Plasma levels of BDNF were higher in SA than in AMI patients (85.7 (19.3–264.4) pg/mL versus 38.6 (10.0–232.2) pg/mL; *p* = 0.0289) (Figure 1). A significant decreasing trend in plasma BDNF levels was observed from HS to SA and to AMI (*p* < 0.0001).

### 3.2. Plasma BDNF and OCT Findings

Table S2 shows coronary plaque characteristics assessed at OCT in the two groups of patients with CAD.

Eighteen (75%) AMI patients had plaque rupture in comparison to SA in whom only eight (27%) patients displayed this characteristic (*p* = 0.001). As expected, AMI patients showed, more frequently, typical features of rupture-prone coronary plaques. In particular, a thin cap fibroatheroma (TCFA) was present in 50% of AMI patients and in 17% of SA patients. Moreover, lipid arc degree and max lipid arc degree were greater in AMI than in SA patients (*p* < 0.001). Intraplaque macrophage infiltration (MØI) was similar in the two groups.

Thirty-two (58%) CAD patients had at least two OCT features of plaque vulnerability.

**Figure 2:**
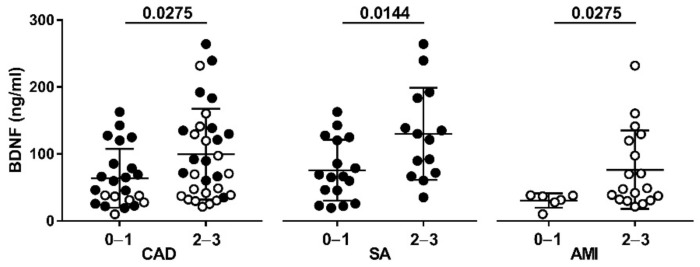
Plasma BDNF levels in CAD patients with diagnosis of SA and AMI in relation to plaque vulnerability core. Plasma BDNF levels were analyzed in relation with low (group 0–1) or high (group 2–3) risk of plaque vulnerability. OCT features of plaque vulnerability were the presence of TCFA, lipid plaque and/or MØI. Data are expressed as mean  ±  SEM.

Patients with a vulnerable plaque, and thus at higher risk of rupture, had higher plasma BDNF levels. In particular, the BDNF levels were approximately two times as high in patients with at least two OCT features of plaque vulnerability, compared to those without, in the overall CAD population (group 2–3: 80.88 (21.44–264.4) pg/mL versus group 0–1: 46.67 (10.01–162.8) pg/mL; *p* = 0.0275), and in patients with SA (group 2–3: 125.8 (35.04–264.4) pg/mL versus group 0–1: 66.32 (19.29–162.8) pg/mL; *p* = 0.0144) and AMI (group 2–3: 48.2 (21.44–232.2) pg/mL versus group 0–1: 34.18 (10.01–38.27) pg/mL; *p* = 0.0275), considered separately (Figure 2). The differences were maintained also after adjustment for prior cardiovascular treatment (*p* < 0.03) and risk factors (*p* < 0.05).

Higher BDNF levels were found in patients with lipid plaque than in those without (*p* = 0.007) and in patients with MØI than in those without (*p* = 0.030) (Table 2) In particular, after adjustment for prior cardiovascular treatment and risk factors, the difference between patients with and without lipid plaque (*p* < 0.005) as well as between patients with and without MØI (*p* < 0.037) was maintained.

**Figure 3:**
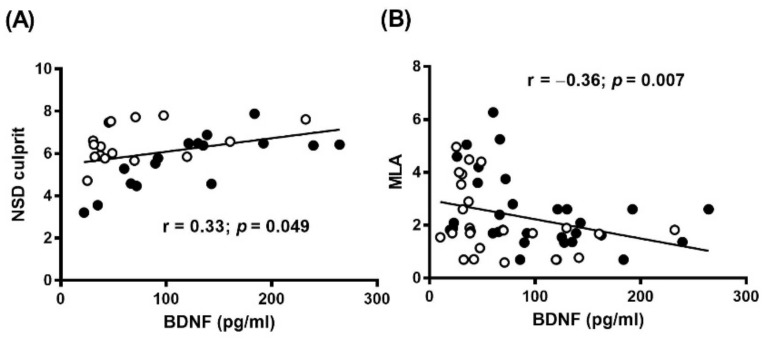
Relation between circulating BDNF levels and OCT features. Correlation between plasma BDNF and (**A**) MØI in terms of normalized standard deviation (NSD) culprit, or (**B**) Minimal Lumen Area (MLA) in CAD patients. SA: black dots, and AMI: white dots.

In addition, BDNF levels correlated positively with MØI (r = 0.33, *p* = 0.049), and inversely with minimal lumen area (MLA: r = −0.36, *p* = 0.007) (Figure 3A,B and Table S3). These differences remained significant even after adjustment for clinical presentation (Table S4), risk factors or prior cardiovascular treatment (NSD: r = 0.52, *p* < 0.002; MLA: r = −0.41, *p* < 0.003).

In the two groups (Table 3), the presence of lipid plaque was associated with higher BDNF levels in both SA and AMI groups (SA: 46.47 pg/mL (22.52–106.1) versus 106.3 pg/mL (66.48–136), *p* < 0.033; AMI: 31.37 pg/mL (18.97–37.41) versus 47.55 pg/mL (32.23–119.8), *p* < 0.024). The difference was maintained in SA group also after adjustment for prior cardiovascular treatment (*p* < 0.026) and risk factors (*p* < 0.015), whereas it was lost in AMI groups after adjustment for diabetes (*p* = 0.221), hypertension (*p* = 0.083) and β-blockers (*p* = 0.052), but was maintained after adjustment for smoke, family history of CAD and dyslipidemia (*p* < 0.044) as well as for prior ACE-inhibitor, Statin and Aspirin treatment (*p* < 0.038).

**Figure 4:**
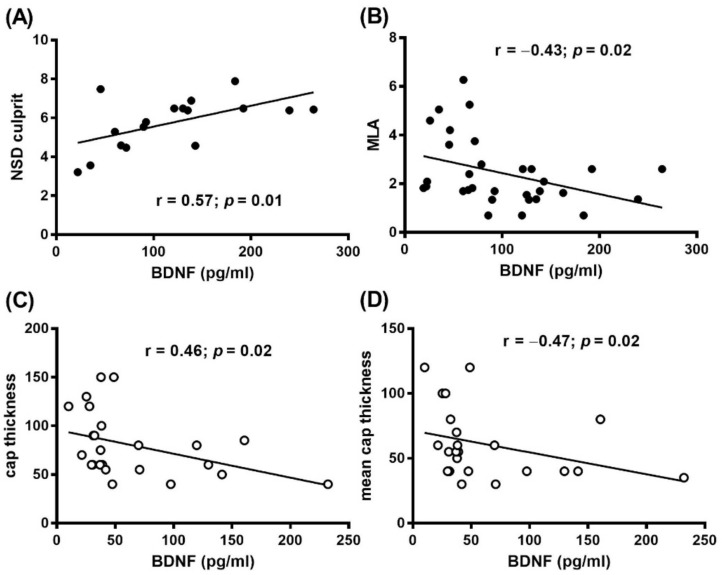
Relation between circulating BDNF levels and OCT features. Correlation between plasma BDNF and (**A**) MØI in terms of normalized standard deviation (NSD) culprit, (**B**) Minimal Luminal Area (MLA), and (**C**) cap thickness and (**D**) mean cap thickness in (**A**,**B**) SA (black dots), and (**C**,**D**) AMI (white dots) patients.

In addition, in SA patients, a positive correlation between BDNF levels and MØI (r = 0.57, *p* = 0.01) and a negative correlation between BDNF and MLA (r = −0.43, *p* = 0.02) were found (Figure 4A,B and Table S5). These differences remained significant even after adjustment for risk factors or prior treatment (NSD: r = 0.58, *p* < 0.02; MLA: r = −0.43, *p* < 0.02) (Table S6).

In AMI patients a negative correlation between BDNF levels and cap thickness (r = −0.46, *p* = 0.02), and mean cap thickness (r = −0.47, *p* = 0.02) was found (Figure 4C and Table S5). This difference persisted even after adjustment for risk factors or prior treatment (cap thickness: r = −0.57, *p* = 0.006), mean cap thickness (r = −0.48, *p* = 0.02) (Table S6).

## 4. Discussion

In this study, we investigated the intriguing link between circulating BDNF and CAD by assessing its relationship with in vivo coronary atherosclerotic plaque morphology. Our main findings were (i) plasma BDNF levels decrease with the presence and clinical acuity and severity of CAD; and interestingly, (ii) higher plasma BDNF levels are closely associated with OCT features of plaque vulnerability in both SA and AMI patients.

By its nature, BDNF is defined as a pro-survival and protective molecule in both neuronal and non-neuronal systems [8]. Indeed, it is expressed in platelets and monocytes as well as in vascular cells [8,18,33], where it plays a key role in regulating processes related to endothelial cells [18] and to vascular smooth muscle cells’ homeostasis and function [9,33,34,35]. Moreover, BDNF has been detected in atherosclerotic plaque and has been found in smooth muscle cells and in macrophages at coronary atheromatous intima and adventitia [19,32,36].

In our study, in agreement with Jin et al., [32] we found that plasma BDNF levels are significantly lower in CAD patients compared to HS. In addition, we observed that, among CAD patients, those hospitalized with AMI have lower BDNF levels than patients with SA. These findings are in agreement with the knowledge that reduced serum levels of BDNF have been associated with a higher risk of cardiovascular event and death, as emerged in the large prospective cohort of the Framingham Heart Study [15].

Thus, it can be hypothesized that BDNF is involved in coronary atherosclerotic plaque progression and activity. However, the behavior of plasma BDNF levels in patients with CAD only and, in particular, in those with SA and AMI, is still controversial. On the one hand, BDNF has been reported to be increased when measured in the first hours after AMI [37], and to be associated with the severity of acute heart failure due to AMI [38]. On the other hand, reduced BDNF levels have been related to adverse cardiac remodeling after AMI [39,40]. Experimental AMI models also provided conflicting results, with some reports showing that BDNF reduces infarct size and ameliorates cardiac function [41], others showing that it increased the extent of inflammation and myocardial injury [42].

Based on this evidence, either a protective or detrimental role of BDNF in CAD has been proposed. However, all these studies stratified BDNF levels according to the clinical presentation of CAD patients (SA and/or AMI), regardless of the morphological features of their underlying coronary plaque. Thus, differences in plaque characteristics might explain the divergent BDNF behavior observed in CAD patients among studies. Notably, in patients with acute coronary syndromes, Montone et al. [25] recently found a close association between high BDNF serum levels and in vivo macrophage infiltration at coronary plaque level, a marker of plaque instability. Whether this applies to all plaque vulnerability characteristics across the clinical spectrum of CAD has not been investigated thus far.

In our group of CAD patients, circulating levels of BDNF were related to coronary plaque vulnerability, as assessed by the in vivo OCT analysis. Indeed, in both SA and AMI patients, those with at least two OCT features of plaque vulnerability had higher BDNF levels. In particular, in SA patients, plasma BDNF levels positively correlated with the infiltration of macrophages, as indicated by NSD culprit, and, negatively, with the MLA, a surrogate of plaque dimension. These intriguing findings could suggest that, facing an increase in vessel stenosis severity, infiltrated inflammatory cells release BDNF. Whether this is aimed at re-equilibrating the system or anticipating plaque rupture, is not known and cannot be inferred from our data. However, previous observations that BDNF preserves from endothelial dysfunction and that low BDNF levels are associated with high von Willebrand Factor levels [32], support the re-equilibrating effect as more likely. Conversely, BDNF increase might have deleterious effects on plaque vulnerability through several mechanisms. Among them, BDNF has been demonstrated to enhance oxidative stress and to promote tissue factor (TF) activity in monocyte/macrophages [19,20]. Indeed, oxidative stress, inducing endothelial dysfunction, lipid oxidation, expression of adhesion molecules and monocyte recruitment, has been considered an important modulator of atherosclerotic plaque progression and vulnerability and has been proposed as a potential therapeutic target of atherosclerotic processes and plaque instability [43]. Moreover, BDNF can induce intra-plaque expression of TF, and enhance inflammation that, in turn, through the activation of TF–FVIIa/PAR-2 pathway, leads to plaque thrombogenicity and vulnerability [44]. Similarly, BDNF could influence plaque remodeling and its instability by activating pro-apoptotic and pro-necrotic pathways and by supporting angiogenesis processes [8,18,19,45,46]. The stronger inflammatory processes, associated with the presence of TF and the release of matrix degrading proteases and of pro-angiogenic compounds, predispose to plaque rupture, and, when combined with the provision of cholesterol, cytokines, and leukocytes in the necrotic core, establish a vicious positive feedback loop for plaque destabilization [47,48,49]. Finally, in the light of the emerging inflammatory atherogenic role of platelets [50], the positive relationship between BDNF and platelet activity in AMI, as evaluated by soluble P-selectin and soluble CD-40-ligand [37], and to thrombus formation [10], might indirectly support the hypothesis of its role in inflammation.

Our data have to be read taking into account that different processes, such as platelets activation, inflammation and endothelial dysfunction, typically present in CAD patients [51,52], can influence or be influenced by BDNF. These factors could be representing a bias or, on the other hand, a cofactor in determining BDNF levels and consequently its relationship with plaque instability.

However, future mechanistic studies are warranted to confirm our preliminary findings and to better elucidate the role of BDNF in coronary plaque activity and stability.

It is important to underlie that the findings of our study have to be interpreted in the context of several limitations. We firstly have to mention that participants were recruited from one center only and that we considered only a small study population, in particular when the comparison was separately conducted in SA and AMI patient groups. Second, circulating BDNF levels are affected by many factors, and not all of the potential determinants were here controlled and measured. In particular, we did not collect information about psychiatric conditions or habitual physical activity that may represent important confounders when circulating BDNF are measured. Third, OCT imaging is a suboptimal reflection of true histology, mainly for macrophage infiltration [26], and one site in an epicardial coronary vessel has been examined. Thus, we cannot exclude that the OCT evaluation of the entire epicardial coronary tree might have helped to better define the association between BDNF and plaque morphology and vulnerability, as assessed by OCT. Fourth, the follow-up of patients is not available, limiting the prognostic value of BDNF detection. Finally, the potential molecular mechanisms explaining the relationship here found have not been investigated.

## 5. Conclusions

The link between plasma BDNF and characteristics of coronary plaques assessed by OCT analysis may suggest a relationship between BDNF and coronary plaque vulnerability. In particular, from our results, although preliminary, it seems that the more vulnerable the plaque, the higher the BDNF levels, regardless of the clinical CAD manifestation. Specific studies investigating the direct association between vessel wall morphology and circulating BDNF in rat animal model will be crucial to disentangle this intriguingly question.

However, further and well-designed investigation, also including clinical follow-up, and conducted in a larger study population, will be important to confirm our results and assess the potential prognostic value of circulating BDNF in plaque vulnerability. In addition, more detailed experiments, with the aim of comprehending the real involvement of inflammatory cells and the molecular mechanisms to explain these relationships, will clarify the true biological meaning of BDNF in coronary atherosclerotic pathology and, hence, in coronary plaque instability.

## Figures and Tables

**Table 1:** Clinical and laboratory data in HS and CAD patients.

Characteristics	HS (*n* = 21)	CAD (*n* = 55)	*p*-Value
Age, years	58.86 ± 12.84	63.05 ± 11.03	0.25
Male sex, *n* (%)	19 (90%)	47 (85.5%)	0.91
BMI, kg/m^2^	28.23 ± 4.01	30.31 ± 4.86	0.19
WBC, 10^3^/μL	5.97 ± 1.26	8.94 ± 3.22	<0.0001
PLT, 10^3^/μL	216.8 ± 49.06	226.1 ± 77.25	0.94
Total cholesterol, mg/dL	223.1 ± 41.03	193.4 ± 43.02	0.01
HDL cholesterol, mg/dL	52.19 ± 17.19	50.6 ± 13.86	0.69
LDL cholesterol, mg/dL	147.5 ± 36.26	113.7 ± 38.27	0.001
Triglycerides, mmol/L	125.9 ± 48.59	146.8 ± 66.11	0.26
Glycaemia, mg/dL	108.3 ± 24.5	137.1 ± 37.58	0.0003
Hb, g/dL	15.43 ± 0.90	13.41 ± 2.52	<0.0001
Smokers, *n* (%)	4 (19.0%)	31 (56.4%)	0.008
Diabetes, *n* (%)	3 (14.3%)	21 (38.2%)	0.08
Hypertension, *n* (%)	8 (38.1%)	31 (56.4%)	0.24
**Drug treatment**			
ACE-inhibitors, *n* (%)	4 (19%)	17 (30.9%)	0.45
Statin, *n* (%)	0 (0%)	19 (34.5%)	<0.0001
β-blockers, *n* (%)	2 (9.52%)	18 (32.7%)	0.08
Aspirin, *n* (%)	0 (0%)	17 (38.9%)	<0.0001

Abbreviations: BMI: body mass index; WBC: white blood cells; PLT: platelet; HDL: high density lipoprotein; LDL: low density lipoprotein; Hb: hemoglobin; ACE; angiotensin-converting-enzyme. Data are presented as number (%) for dichotomous variables, mean ± standard deviation for continuous variables with a normal distribution and median (interquartile range [IQR]) for non-parametric continuous variables.

**Table 2:** Plasma BDNF levels according to OCT features in CAD patients.

OCT Parameters	BDNF (pg/mL)		*p*-Value
	No	Yes	
Thrombus	75.38 (38.43–130.1) (*n* = 40)	41.94 (30.72–70.85) (*n* = 15)	0.05
Plaque rupture	78.83 (42.91–140.1) (*n* = 29)	46.04 (32.02–103.2) (*n* = 26)	0.15
Lipid plaque	37.41 (22.74–66.27) (*n* = 14)	78.83 (40.43–130) (*n* = 41)	0.004
TCFA	65.78 (32.02–125.8) (*n* = 38)	69.98 (40.43–135.6) (*n* = 17)	0.32
Micro-vessel	66.43 (31.21–131.4) (*n* = 34)	69.98 (37.84–120.1) (*n* = 21)	0.64
Multivessel	46.47 (33.63–123.3) (*n* = 21)	69.66 (40.92–131.4) (*n* = 34)	0.39
NSD culprit	53.16 (27.43–94.41) (*n* = 22)	71.92 (39.89–140.9) (*n* = 33)	0.03

Abbreviations: TCFA: Thin-cap fibroatheroma; NSD: Normalized standard deviation. Data are presented as median (interquartile range (IQR)).

**Table 3:** Plasma BDNF levels according to OCT features in SA and AMI patients separately.

OCT Parameters	BDNF (pg/mL)		*p*-Value
	No	Yes	
SA patients			
Thrombus	85.73 (59.84–135.1) (*n* = 31)	(*n* = 0)	.
Plaque rupture	78.83 (59.84–138.8) (*n* = 23)	106.8 (51.49–129.5) (*n* = 8)	0.58
Lipid plaque	46.47 (22.52–106.1) (*n* = 9)	106.3 (66.48–136) (*n* = 22)	0.01
TCFA	82.28 (49.92–137.9) (*n* = 26)	85.73 (59.84–120.5) (*n* = 5)	0.27
Micro-vessel	84.33 (31.21–131.4) (*n* = 24)	69.98 (37.84–120.1) (*n* = 7)	0.82
Multivessel	106.8 (43.61–139.8) (*n* = 6)	78.83 (60.06–137) (*n* = 25)	0.81
NSD culprit	66.32 (36.22–103.1) (*n* = 13)	125.8 (64.97–168) (*n* = 18)	0.04
AMI patients			
Thrombus	38.27 (30.62–124.8) (*n* = 9)	41.94 (30.72–70.85) (*n* = 15)	0.74
Plaque rupture	88.65 (31.21–164.1) (*n* = 6)	37.63 (30.51–70.2) (*n* = 18)	0.29
Lipid plaque	31.37 (18.97–37.41) (*n* = 5)	47.55 (32.23–119.8) (*n* = 19)	0.03
TCFA	34.82 (25.98–46.2) (*n* = 12)	58.77 (37.47–121.7) (*n* = 12)	0.07
Micro-vessel	34.61 (29.38–61.03) (*n* = 10)	40.43 (35.79–122.3) (*n* = 14)	0.69
Multivessel	38.27 (29.86–119.8) (*n* = 15)	41.94 (31.05–70.42) (*n* = 9)	0.92
NSD culprit	36.98 (24.68–84.33) (*n* = 9)	47.55 (32.23–97.6) (*n* = 15)	0.21

Abbreviations: TCFA: Thin-cap fibroatheroma; NSD: Normalized standard deviation. Data are presented as median (interquartile range (IQR)).

## Data Availability

Data are available within the article and/or on-line Supplementary Materials.

## Supplementary Materials

The following are available online at https://www.mdpi.com/article/10.3390/diagnostics11061010/s1, Table S1: Clinical and laboratory data in HS and CAD patients; Table S1: Clinical and laboratory data in SA and AMI patients; Table S2: Coronary plaque characteristics assessed at OCT in SA and AMI patients; Table S3: Predictors of BDNF in CAD patients; Table S4: Multiple regression predictors of BDNF in CAD patients after adjustment for sex, age and AMI presentation; Table S5: Univariate predictors of BDNF in SA and AMI patients; Table S6: Multiple regression predictors of BDNF in SA and AMI patients after adjustment for sex and age.
