# Supplementary material for: Potential Relation between Plasma BDNF Levels and Human Coronary Plaque Morphology
Source: Diagnostics (Basel). 2021 Jun 1;11(6):1010. doi: 10.3390/diagnostics11061010 (PMC8226920; doi:10.3390/diagnostics11061010)
Supplement: Supplementary file 1 [file diagnostics-11-01010-s001.zip › diagnostics-1214703-supplementary.pdf]

Supplementary Material

Table S1. Clinical and laboratory data in HS and CAD patients.

Characteristics	HS (N = 21)	CAD (N = 55)	P-value
Age, years	58.86 ± 12.84	63.05 ± 11.03	0.25
Male sex, N (%)	19 (90%)	47 (85.5%)	0.91
BMI, Kg/m^2	28.23 ± 4.01	30.31 ± 4.86	0.19
WBC, 10^3/μL	5.97 ± 1.26	8.94 ± 3.22	**<0.0001**
PLT, 10^3/μL	216.8 ± 49.06	226.1 ± 77.25	0.94
Total cholesterol, mg/dL	223.1 ± 41.03	193.4 ± 43.02	**0.01**
HDL cholesterol, mg/dL	52.19 ± 17.19	50.6 ± 13.86	0.69
LDL cholesterol, mg/dL	147.5 ± 36.26	113.7 ± 38.27	**0.001**
Triglycerides, mmol/L	125.9 ± 48.59	146.8 ± 66.11	0.26
Glycaemia, mg/dL	108.3 ± 24.5	137.1 ± 37.58	**0.0003**
Hb, g/dL	15.43 ± 0.90	13.41 ± 2.52	**<0.0001**
Smokers, N. (%)	4 (19.0%)	31 (56.4%)	**0.008**
Diabetes, N (%)	3 (14.3%)	21 (38.2%)	**0.08**
Hypertension, N (%)	8 (38.1%)	31 (56.4%)	0.24
**Drug treatment**			
ACE-inhibitors, N (%)	4 (19%)	17 (30.9%)	0.45
Statin, N (%)	0 (0%)	19 (34.5%)	**<0.0001**
β-blockers, N (%)	2 (9.52%)	18 (32.7%)	0.08
Aspirin, N. (%)	0 (0%)	17 (38.9%)	**<0.0001**

Abbreviations: BMI: body mass index; WBC: white blood cells; PLT: platelet; HDL: high density lipoprotein; LDL: low density lipoprotein; Hb: hemoglobin; ACE; angiotensin-converting-enzyme. Data are presented as number (%) for dichotomous variables, mean ± standard deviation for continuous variables with a normal distribution and median (interquartile range [IQR]) for non-parametric continuous variables.

Table S2. Clinical and laboratory data in SA and AMI patients.

Characteristics	SA (N = 31)	AMI (N = 24)	P-value
Age, years	65.29 ± 9.98	60.17 ± 11.84	0.16
Male sex, N (%)	26 (83.9%)	21 (87.5%)	0.99
BMI, Kg/m^2	29.90 ± 4.92	30.83 ± 4.80	0.46
RBC, 10^6/μL	4.45 ± 0.56	5.15 ± 2.24	0.08
WBC, 10^3/μL	8.90 ± 3.07	9.00 ± 3.47	0.92
PLT, 10^3/μL	222.5 ± 68.81	230.6 ± 88.31	0.57
Neutrophils, 10^3/μL	5.85 ± 2.61	5.84 ± 3.25	0.80
Eosinophils, 10^3/μL	0.46 ± 1.30	0.33 ± 0.47	0.32
Basophils, 10^3/μL	0.02 ± 0.04	0.02 ± 0.07	1.00
Lymphocytes, 10^3/μL	2.0 (1.6;3.1)	1.90 (1.3;2.88)	0.41
Monocytes, 10^3/μL	0.64 ± 0.24	0.65 ± 0.26	0.94
Hb, g/dL	13.47 ± 1.76	13.34 ± 3.29	0.49
Total cholesterol, mg/dL	181.2 ± 36.2	209.2 ± 46.63	**0.01**
HDL cholesterol, mg/dL	54.9 ± 15.21	45.05 ± 9.59	**0.009**
LDL cholesterol, mg/dL	101.1 ± 27.5	130 ± 44.29	**0.002**
Triglycerides, mmol/L	125 ± 63.78	174.9 ± 59.06	**<0.001**
Glycaemia, mg/dL	125.9 ± 26.78	151.5 ± 44.65	**0.03**
Urea, mg/dL	47.81 ± 15.99	43.17 ± 17.97	0.10
Creatinine, mg/dL	0.95 ± 0.24	1.01 ± 0.42	0.67
Uric acid, mg/dL	4.92 ± 1.05	5.25 ± 1.98	0.49
CRP , mg/L	2.1 (1;2.8)	9.80 (2.88;21.0)	**<0.001**
Tnl, ng/mL	0 (0;0.01)	5.68 (1.33;37.84)	**<0.001**
Ejection Fraction, %	53.68 ± 7.29	48.87 ± 9.15	**0.04**
Smokers, N. (%)	18 (58.1%)	13 (54.2%)	0.99
Diabetes, N (%)	9 (29.0%)	12 (50.0%)	0.16
Hypertension, N (%)	19 (61.29%)	12 (50.0%)	0.57
Familiarity, N (%)	12 (38.71%)	15 (62.50%)	0.14
Dyslipidemia, N (%)	15 (48.39%)	13 (54.2%)	0.50
**Drug treatment**			
ACE-inhibitors, N (%)	13 (41.96%)	4 (16.67%)	0.09
Statin, N (%)	14 (45.16%)	5 (20.83%)	0.11
β-blockers, N (%)	11 (35.48%)	7 (29.17%)	0.89
Aspirin, N. (%)	10 (32.26%)	7 (29.17%)	0.82

Abbreviations: BMI: body mass index; RBC: red blood cells; WBC: white blood cells; PLT: platelet; Hb: hemoglobin; HDL: high density lipoprotein; LDL: low density lipoprotein; CRP: C-reactive protein; TnI: Troponin I; ACE; angiotensin-converting-enzyme. Data are presented as number (%) for dichotomous variables, mean ± standard deviation for continuous variables with a normal distribution and median (interquartile range [IQR]) for non-parametric continuous variables.

Table S3. Coronary plaque characteristics assessed at OCT in SA and AMI patients

OCT parameters	SA (N = 31)	AMI (N = 24)	P-value
Plaque rupture, N (%)	8 (25.8%)	18 (75%)	**<0.001**
Lipid plaque (%)	21 (67.7%)	19 (79.2%)	0.53
TCFA, N (%)	5 (16.1%)	12 (50%)	**0.02**
Cap thickness	95.81 ± 24.12	80.0 ± 33.1	**0.03**
Min cap thickness	77.67 ± 17.67	60.87 ± 27.21	**0.005**
MLA	1.89 (1.54;2.8)	1.78 (1.24;3.83)	0.75
Lipid arc degree	125 (65;265)	265 (149.5;280)	**0.001**
Max calcified arc	84 (49;135)	79.75 (60;130.5)	0.77
Max lipid arc	159 (86;280)	227(206;300)	**<0.001**
Stenosis, %	70.67 ± 19.96	76.85 ± 18.38	0.06
Microvessel (%)	7 (22.6%)	14 (58.3%)	**0.015**
Multivessel (%)	25 (58.06%)	9 (37.5%)	**0.003**
NSD culprit (%)	18 (75%)	15 (62.5%)	0.23
NSD culprit	5.74 ± 1.28	6.43 ± 0.99	0.15

Abbreviations: TCFA: Thin-cap fibroatheroma; MLA: minimal luminal area; NSD: Normalized standard deviation. Data are presented as number (%) for dichotomous variables, mean ± standard deviation for continuous variables with a normal distribution and median (interquartile range [IQR]) for non-parametric continuous variables.

Table S4. Predictors of BDNF in CAD patients.

OCT parameters	r	P-value
Cap thickness	-0.04	0.76
Min-cap-thickness	-0.17	0.28
MLA	**-0.33**	**0.02**
Lipid arc degree	-0.11	0.44
Max calcified arc	-0.03	0.84
Max lipid arc	-0.24	0.08
Stenosis	0.09	0.47
NSD culprit	**0.37**	**0.04**

Abbreviations: MLA: minimal luminal area; NSD: Normalized standard deviation

Table S5. Multiple regression predictors of BDNF in CAD patients after adjustment for sex, age and AMI presentation.

OCT parameters	r	P-value
Cap thickness	-0.12	0.40
Min-cap-thickness	-0.19	0.25
MLA	**-0.34**	**0.02**
Lipid arc degree	-0.01	0.66
Max calcified arc	-0.02	0.87
Max lipid arc	-0.19	0.18
Stenosis	0.10	0.48
NSD culprit	**0.52**	**0.03**

Abbreviations: MLA: minimal luminal area; NSD: Normalized standard deviation

Table S6. Univariable predictors of BDNF in SA and AMI patients.

OCT parameters	r	P-value
**SA patients**		
Cap thickness	0.08	0.66
Min-cap-thickness	-0.32	0.16
MLA	**-0.43**	**0.02**
Lipid arc degree	0.10	0.59
Max calcified arc	0.01	0.94
Max lipid arc	-0.09	0.62
Stenosis	0.27	0.15
NSD culprit	**0.57**	**0.02**
**AMI patients**		
Cap thickness	**-0.47**	**0.02**
Mean-cap-thickness	**-0.40**	**0.049**
MLA	-0.26	0.23
Lipid arc degree	0.00	0.99
Max calcified arc	-0.19	0.38
Max lipid arc	-0.15	0.47
Stenosis	0.14	0.51
NSD culprit	0.28	0.29

Abbreviations: MLA: minimal luminal area; NSD: Normalized standard deviation

Table S7. Multiple regression predictors of BDNF in SA and AMI patients after adjustment for sex and age.

OCT parameters	r	P-value
**SA patients**		
Cap thickness	0.14	0.46
Min-cap-thickness	-0.30	0.15
MLA	**-0.43**	**0.02**
Lipid arc degree	0.06	0.74
Max calcified arc	0.05	0.79
Max lipid arc	-0.08	0.67
Stenosis	0.26	0.18
NSD culprit	**0.55**	**0.04**
**AMI patients**		
Cap thickness	**-0.57**	**0.01**
Mean-cap-thickness	**-0.50**	**0.02**
MLA	-0.17	0.44
Lipid arc degree	0.17	0.43
Max calcified arc	-0.05	0.81
Max lipid arc	-0.29	0.19
Stenosis	0.05	0.84
NSD culprit	0.24	0.42

Abbreviations: MLA: minimal luminal area; NSD: Normalized standard deviation.
